# Extracts and Gleanings from Our Exchanges

**Published:** 1873-10

**Authors:** 


### PART II.

### EXTRACTS AND GLEANINGS FROM OUR EXCHANGES.

### *MULTUM IN PARVO.*

*Mess. Editors,*—I was called August 23, to see Mrs. Price, who is stout and healthy, about 25 years old. Upon entering the room I found her in a terrific convulsion, caused by a large dose of strychnia. Her whole person seemed to be participating in the spasm; hands tightly clenched, legs and arms were flexed and extended alternately with great power, jaws closely and very firmly locked. It was sometimes a minute or two after the other spasms had ceased before the mouth could be opened, then she swallowed with difficulty.

As soon as possible I administered twenty grains of the sulphate of zinc in solution, and sent for a stomach pump, chloroform, and the saturated tincture of calabar bean.

The chloroform arriving some time first, I commenced its use by inhalation, trying to control the spasms, but with only partial effect, as I did not want to get her thoroughly under the influence of the drug.

Gave as much muc. g. acacia as she would drink. Repeated the zinc in about fifteen minutes from the time the first was taken; it caused free vomiting.

The convulsions continued to return every five minutes, lasting about one minute, but with less severity—thanks to the chloroform, which she soon appreciated, as demonstrated by calling for it, when she felt one coming on.

The stomach pump was used promptly upon its arrival, and the stomach thoroughly washed out with warm water.

I then put three drops of the tincture of calabar bean in fifteen of water, and gave it hypodemically. Just as I took the needle from the arm she had one of the most severe spasms, but it was the last of the kind, each succeeding one being of less force, until they ceased entirely, and the lady recovered without a bad symptom.

The next day I questioned her very closely as to the amount taken. She said, "I bought that vial full," (a drachm vial with not more than twenty grains left in it), "but did not know how much it would take to kill me. I first put some on a buttered biscuit and ate it, as that did not kill immediately, put some more on a slice of watermelon and ate it, but I vomited shortly after that;" (she did not however throw it all up, as was evidenced by the egesta brought up by the zinc and pump), "this failing too, I put a teaspoonful in some whisky and water and drank all except what was left in the glass." The glass referred to was about a fourth full of the mixture; upon pouring it off, five or ten grains were found at the bottom of the glass. So she must have taken about 30 grains of strychnia.

She thinks it was over half an hour between taking the first and last doses. The pain caused by the convulsions aroused her neighbors, who sent for me. They thought it half an hour before I arrived, and it was certainly longer than that before the stomach pump was used; making an hour and a half after the taking of the first dose, and an hour after the last, which is ample time for a fatally poisonous quantity to have been absorbed into the system.

The question now is, what antidoted the strychnia already in the system at the time the stomach pump was used? The pump merely prevented the absorption of any more, but could not have had any effect upon that then causing the terrible convulsions. As the chloroform had failed to produce such an effect up to that time, I am forced to believe the good result is due to the calabar bean.

Yours respectfully, E. T. HENRY.

*Vicksburg, Sept.* 11, 1873.

*Mess. Editors,*—On June 21st, 1873, Jesse Vaughan, aged 8 or 9 years, son of H. D. Vaughan, accompanied his father to the gin-house, to have some corn ground. The machine is worked by horse-power. The band-wheel is only 3 inches above the lever. It seems that the boy lay on the lever with his back to the wheel, and as the lever was brought under the wheel, his head was caught between the lever and wheel. The team attached to the lever was stopped by the boy's head as it was drawn under the band-wheel. When the father ran down to see what was the matter, he found his child's head between the lever and band-wheel, fast and flattened. The lever could not be reversed. In order to extricate the head, he had "to whip up the mules and run the head through." I saw the child six hours after the accident. It was still alive, pulse 140, breathing quick. The scalp, from the eyebrows to the crown of the head and behind the ears was loose from the skull. All that part of the skull from an inch behind the ears to the crown and front, was crushed. The back and base were not injured. The blood ran freely from the mouth and nose at first.

In this condition the boy lived eight hours. Just before he died he turned his head and drew up his arms and legs.

I did not make a post mortem of the head, but judging from the size of the head and the narrow space it passed through, being but three inches, the entire front head and face were broken and crushed.

I report this case to show how long a person may live after nearly the entire cerebrum was mashed into a pulp, with the cerebellum uninjured. A. S. HELMICK, M. D.

*Bustrop, Morehouse Parish, La.*

Remedy for Tape-Worm.—Mrs. B., aged about thirty years, called at my office in May, and stated that she had been troubled for two years with tape-worm. She had expelled parts of the worm on different occasions. She presented that peculiar appearance always attending one promising of tape-worm. I ordered her to eat no supper nor breakfast. Called at 9 A. M. the next morning, and finding she had fasted, gave her half the following mixture:

℞: Gum arabic, ʒ ij; turpentine-water, aa f ℥ j; sweet milk, f ℥ ij; oil cinnamon, ℈ ij.

Waited one hour; no effect save slight nausea. Gave the remaining half of turpentine mixture, followed in thirty minutes by half ounce castor oil. One hour from the administration of the oil the patient expelled fifteen feet of worm, together with the head. The only disagreeable effect from so large a dose of turpentine was nausea and slight intoxication, which gradually wore off, and was gone in four hours.

After expulsion of the worm, I ordered twenty drops camph., tr. opium in mint water. Ordered half ounce castor oil every morning for three, and left. My patient is now in good health.

W. W. HARRISON.

*Alexandria, Ala., July* 29, 1873.

Headache in Children.—At a recent meeting of the Harveian Society, of London, Dr. W. H. Day read a paper on Headache in Children. After remarking on the symptomatic value of headache, as a term, the author reviewed the commonest variety of headache in children, and then dwelt especially on a peculiar functional form, of which he had met with several examples. These were associated, he believed, with some intricate, though undemonstrable, change, physical, structural or chemical, going on within the cranium, giving rise to some confusion or abnormal sensation in the head, or to actual headache. This peculiar cerebral change is accompanied occasionally, but not of necessity, with enlargement of the head, wasting of the extremities, flabbiness of the muscles, and looseness of the joints; there are also pallor and debility, restlessness by night, irritability by day, a slow and sometimes irregular pulse, with dull and persistent headache; the temperature is normal. Dr. Day treated the early stage of the disease with bromide and iodide of potassium; and advocated later on the use of iron, bark, and cod-oil. Tonics did harm in the early treatment.

Dr. Farquharson gave some interesting experience in relation to headache in children, based upon cases treated by him at Rugby. He referred to the importance of not overlooking headache as a symptom of slight sunstroke, and in connection with over-work; in the latter cases, the urine contained alkaline phosphates.

Dr. Broadbent regarded persistent headache in a child as a suspicious symptom. Dr. Day's cases he considered examples of the headache of rickets or hydrocephalus. He advocated the use of small doses of mercury continued for a long time.—*Medical and Surgical Reporter.*

Digitalis in Dropsy.—By G. C. Pitzer, M. D., Detroit, Ills., (Eclectic Med. Jour.) writes :

During the Summer and Fall of 1865, we had an epidemic of scarlet fever in this county, and a great many of the cases were very severe. I treated it almost exclusively with belladonna, and with uniform success, but a few of the cases were followed with dropsy, which proved to be the most troublesome feature of the disease. Amongst others, my little girls were attacked, and the eldest, then eight years old, had all the symptoms of scarlatina anginosa, followed by general dropsy. For this dropsical condition we resorted to all the best means recommended by the profession without any benefit. We called counsel, changed the treatment from time to time, but without any change in the symptoms for the better. We had made up our minds to give up the case, and had in our own imagination bidden our loved one farewell. About this time digitalis was suggested, and with but little confidence I prepared an infusion of foxglove, as follows:

℞ Foxglove (leaves), ʒj.
Wild Cherry bark, (green), ℥j.
Juniper berries, ℥j. M.

Make half a pint of infusion by adding boiling water, and keeping it nearly boiling hot for an hour, then strain and sweeten with crushed sugar, and when cool add half a pint of best Holland gin.

Of this preparation I gave my little girl one teaspoonful every six hours. The spoon we used in this case held about a drachm and a half, and after the second dose we discovered quite a change in our patient for the better. Now imagine our feelings. From a state of gloom, darkness and despair, we thought we saw a ray of hope. We continued the treatment, and in two days' time the symptoms for the better were marked; and without further trouble or additional means our patient made a rapid recovery. Now this was no little case of a few days' standing. Not at all. For days and weeks we tried in vain to overcome this tendency to dropsy, and many times during the progress of the disease it seemed as though dissolution was inevitable.

While speaking of digitalis, I will instance another case or two, and make no farther reference to dropsical cases following scarlet fever, of which I could report several.

In 1867, a young lady from this neighborhood went to Ohio on a visit to some of her friends, and while there was taken seriously ill, the more prominent symptoms of the case being anasarca—general dropsy. Remember dropsy is a symptom, not a disease. She was treated regularly. The report came back to her friends that she could not, in all probability, recover. Her mother came to me for advice. I remarked that it was impossible for me to determine the particular lesion upon which the dropsy depended, but that if all the usual means had been resorted to without benefit, and she so requested, I would make her a prescription, stating at the same time that we could, in all probability, do her some good. The old lady was very anxious, and having implicit confidence in our skill in this relation, requested me to fix up the prescription at once. I accordingly wrote out the formula as above given, digitalis, wild cherry, juniper berries, etc., and the mother sent it out by the first mail, requesting her friends, in the accompanying letter, to procure the remedies immediately, and give them according to directions, and that she would be there in a short time to explain the matter. In a few days the old lady started, and when she arrived at the residence of her friends she found that they had received her instructions, and were already using the remedies. The young lady was quite sick, but they had implicit confidence in their medicine, and in a few days the symptoms for the better became apparent, and without further difficulty or additional means this case made a rapid recovery.

About six weeks ago, while at the hotel in our county seat, I met an old friend—not a relative or former patron—who resides about two miles from me, and during our conversation he related to me a peculiar difficulty under which he had been laboring for many years. To begin with, I'll just state that this man is about forty-five years of age, bilious lymphatic temperament, temperate habits, weighed at that time 275 pounds, and withal, he is a man of very superior intellect. His statement was about this:

"Doctor, I feel quite unwell. I've not been well for several years; sometimes feel better, sometimes worse; I'm growing more fleshy every year; I've been troubled with some kind of kidney disease for a long time; anti-bilious remedies scarcely give me temporary relief, and leave me worse than they found me; the least exertion makes me puff and blow like a wind-broken horse; I feel dull and stupid all the while—have no energy as I used to have."

He said considerable about his condition not necessary to mention here, after which I gave him an examination and said:

"Mr. H., I'll tell you what you need. You need just one remedy. It won't cost you much, and if you will use it you shall have the benefit of my judgment in your case gratis."

He said, "Well, Dr., what is it?"

I answered, "You need digitalis."

"Digitalis!" said he, "why do you prescribe that?"

I responded, "To meet the indications in your case, sir, and my word for it, if you will procure the remedy in the form of the officinal tincture, and take it in doses of ten to fifteen drops three times a day, you'll receive more benefit from its use than from all the medicine you've taken for ten years."

He went straightway to the drug store, and bought an ounce of the tincture, and commenced using it at once. He called at my office yesterday, and said, "Well, Dr., that digitalis did the work for me, and I've told more than twenty doctors about it; (by the way, Mr. H. is quite a business man and is around considerably,) and now, sir, if you believe me, in thirty days from the time I commenced using the remedy, I had got rid of twenty-eight pounds of my surplus, and I felt better every day from the beginning, and now feel splendid—better than I have felt for years, and you see my clothes that would barely meet on me are entirely too large, and now, Dr., I can run all over this farm with you without the least inconvenience. It is almost incredible but actually so, that a great work has been wrought in my case, and I feel like a new man."

Croton Chloral in Painful Affections of the Fifth Nerve.—It is perhaps surprising that a remedy whose action was several months ago declared to be of so extraordinary a character should have received so little attention at the hands of the profession, especially when this new medicine promised to be so efficient a weapon against some of the most painful diseases known to physicians. Beyond one or two pharmacological notices, the substance seems to have been altogether passed by.

The hydrate of croton chloral was made by Kramer and Pinner, by the action of alkalies upon dichlorallyl and formic acid. Its physiological action was investigated by O. Liebrech. He found that in animals it produced a deep anæsthesia of the head, without any loss of sensibility of the body. Death was caused by a paralysis of medulla oblongata. In man, an anæsthesia of the fifth nerve only was noticed. The sensibility of the trunk, and the pulse and respiration, remained unaltered.

Having procured some of this substance, I determined to make observations upon such of my patients at St. Bartholomew's as appeared likely to be benefitted by the medicine. I gave it to about twenty persons, nearly all women. They varied in age from seventeen to forty-four. They were all suffering pains in the region supplied by the fifth nerve,—that is the upper and lower jaw, the face, and the supra-orbital region of the forehead. The pains were paroxysmal. In the majority of the cases they were increased at night. In nearly every one of these cases there were caries of the teeth. In about half there were signs of anæmia. The medicine was given in doses of five, ten and twenty grains, dissolved in water. It was given at night, just before going to bed. In one case, where the pains became aggravated at noon and at bed-time, it was given just before the increase of pain was expected. In all the patients, except two, great relief from pain followed the dose of croton chloral. Some of the patients said they slept well after it; others, that they did not sleep, but that the pains in the head and face either ceased altogether, or were much diminished. In two cases, both women, the croton chloral was of no use whatever, the pains being aggravated during the use of the medicine; but in the rest of the cases more or less relief was given.

Should the croton chloral be as efficient in the hands of others as it has been in mine, it will prove a most important addition to the materia medica. It will enable the physician to give relief from pain until relief can be afforded by the dentist, or by attention to the general health, and this without any of the general effects of narcotics.—D. J. W. Leg—*Dental Cosmos, Feb., '73,* from *The Lancet.*

PEPSIN.—Several years ago Von Wittich made the discovery that glycerine would extract the peculiar animal ferments of certain organs, such as pepsin from the tissues of the stomach, and pancreatine from the pancreas. Prof. Mitchell Foster repeated these experiments, and found them substantially correct. Since then many experimenters have obtainted pepsin in the same manner.

Wittich's process is to thoroughly clean the stomach of the pig, and strip off the serous coat. The part most rich in pepsin is that between the cardiac and pyloric orifices. After cleaning off the adherent mucus, the tissues are thoroughly dried between paper, or cloth, to absorb all the moisture that can be gotten out. The tissue is then finely minced, and put in a glass jar and covered with glycerine, and set aside for 24 hours, occasionally stirring. The glycerine is then drained off, and another portion is poured on and treated as before. The second is nearly as strongly charged with pepsin as the first. It is not advisable to continue the process, as the third and fourth extracts are not largely charged with pepsin. However, if the glycerine is good (German glycerine will answer), the first extract will be quite or nearly saturated with pepsin, and the second but little inferior. Ten drops of this glycerine extract of pepsin in an ounce of water, and one drop of hydrochloric acid, is worth more than 80 or 100 grains of the dry pepsin in market. Certainly it is more reliable in any event. This extract may be kept a year in a well-closed, glass-stoppered bottle in the dark and a cool place.

The great value of pepsin, as a means of combating the wasting and acute diseases of both infants and adults, is too well appreciated to need one word said in recommending its use. But the swindles practiced on the profession by manufacturers in their dealing out the vile trash, they style pepsin, has made many persons lose confidence in this valuable remedial agent.

Our junior brethren will find most gratifying results accrue to their patients, and add to their own reputation by treating many forms of chronic diseases with pepsin, good food, and hygienic measures, rather than by drug medication. A great many cases of chronic neuralgias, skin diseases—many forms of nervous disorders, the very perplexing and vexatious forms of female diseases, and a host of the diseases of children, will be found more manageable in this way of treating them. Thousands of cases of cholera infantum prove fatal from inanition every year, which might be saved by pepsin and the glycerine pancreatic extract, prepared in the same way as the pepsin. In continued fevers, after the fourteenth day, the mortality increases rapidly. The cause lies in the low assimilating powers of the nutritive system at this period. Here valuable aid may be derived from these glycerine extracts of these important animal ferments, to assist the weakened nutritive forces in supplying pabulum for the blood and tissues. These suggestions are worth the attention of our younger brethren everywhere.

Some physicians say they have obtained a good pepsin wine by adding sherry wine to the mass after the second glycerine extract had been drained off. Twice the bulk of sherry wine is poured upon the mass, and allowed to digest for two or three days, with occasional agitation. The wine is then poured off, the mass pressed, and the fluid strained. The dose of this wine is from one to two teaspoonsful after each meal. The glycerine extract is administered in the proportion of from two to ten drops in water, slightly acidulated with hydrochloric acid, after each meal.—*Medical Archives.*

Carbolic Acid in Treatment of Lupus.—Dr. Chapman reports, in the *N. Y. Medical Review,* a case of lupus of twenty-five years' duration successfully treated by the application of carbolic acid. The patient was aet. sixty-five. The disease had commenced as a small tubercle near the tip of the nose, and, although treated by a number of physicians, had gradually spread to the forehead and cheeks. When seen by Dr. C., erysipelas was setting in in connection with it, and it was spreading rapidly, involving the whole nose, and extending to the cheeks and upper lip. He says:

First of all, to subdue the erysipelatous inflamation, I ordered the whole suface to be penciled once in three hours with the fluid ext. of veratrum viride, and in forty-eight hours had the satisfaction of seeing the erysipelas entirely removed. I then commenced the use of carbolic acid, the remedy with which I expected to cure the disease. I used Nickel's caystals melted, one part, and olive oil, one part; with this I penciled the whole surface of the disease twice a day. Occasionally I would use the clear crystals once a day, before using the carbolated oil. I treated my patient in this way for forty-eight days, when I discharged him cured.

With the experience I have had with the carbolic acid in this disease I should not hesitate to pronounce a cure in any and all cases of lupus. I should use the acid, however, full strength as often as once in two or three days, and not reduce it more than one-half at any time. I prefer reducing it with olive oil, as that seems to keep moist longer than glycerine or anything else, thereby keeping up the action of the remedy longer. How the carbolic acid cures this disease I will leave for wiser heads than mine to discuss. I am quite well satisfied, for the present, to know that it will cure it. During the treatment of this case I gave internally the comp. syr. stillingia with iod. pot., but I claim very little, if any, aid from the constitutional treatment.—*Medical Archives.*

Gonorrhœa, Gleet, etc.—We have recently known a number of very obstinate cases of gleet relieved by the introduction of a catheter, smeared with mild zinc ointment, once or twice per day. Many recent cases of gonorrhœa are much relieved by the same means, with the addition of a little carbolic acid, sulphate of zinc or nitrate of silver. An injection, containing about 2 grs. of sulphate of zinc to the ounce of water, and the whole made thick as cream, with finely-powdered goldenseal (Hydrastis Canadensis), is deemed worth from $500 to $1000 by those who have been very speedily cured by it.—*Med. Times.*

Catarrh.—Dr. Noyes said he was much pleased with the action of stimulant in acute catarrh. He never knew them to fail, though, for obvious reasons, it was not wise always to recommend them to all. Other sedatives, as quinine or opium, accomplished the same end much more safely. The treatment of chronic catarrh by nitrate of silver has been extensively used and abused. Dr. Green, of New York, is said to have passed probangs wet with strong solutions (40 to 80 grains to ounce) into the trachea. He had passed the probang himself, but it was a difficult operation. As in the eye, nitrate of silver excites a new inflammation in the follicles of the throat, which carries off the neoplastic formation. In his specialty he often was required to treat the results of chronic catarrh, for it may extend to the Eustachian tube—the middle ear—to the formation of abscess, or even perforation of tympanum. He had tried all methods, douches, powders, vapors, etc. The douche needs to be used with caution, or the Eustachian will be flooded, and inflammation of the middle ear ensue. This method is especially unsafe for the laity to use.

Dr. Gilbert said that it was very important to perfectly cleanse the membrane ere making any application to it. A favorite local remedy with him was chloride of zinc, one grain to the ounce. Constitutionally he gave stillingia, combined with the iodide of potassium.

Dr. Carstens had tried all the local applications for chronic catarrh, but found none so efficient as glycerine. Internally he used a combination of iodide of potassium and chloride of ammonium.

Dr. Jenks said that in acute catarrh he prescribed small doses of morphia (gr. 1-20th to 1-30th) with great satisfaction.—*Detroit Academy of Medicine.—Detroit Review of Medicine.*

Ammonia in Suspended Animation.—The value of the injection of ammonia, as recommended by Professor Halford, in cases of snake bite and suspended animation, has been again demonstrated. A lady in Melbourne recently swallowed by an accident an ounce of Browne's chlorodyne, which is a mixture of chloroform, morphia and prussic acid. When seen by her medical attendant, she was, as he imagined, on the point of death, cold, insensible to everything, and giving only occasionally gasps as signs of breathing. Recollecting a former case, in which a young man who had taken chloroform was revived after death had apparently occurred, the doctor mixed half a drachm of the liq. ammon. fort. with one and a half of water, and within the space of one minute injected the whole into a vein of the arm. In a few minutes the pulse returned, the breathing became natural, and in twenty minutes the whole body had regained its natural warmth; but perfect consciousness did not return for some hours afterwards. The patient made a rapid recovery. Two further instances have been reported in which the timely use of the injection saved the victims of snake bites from the death which threatened them.—*Melbourne Argus.—N. Y. Med. Jour.*

Treatment of Cancrum Oris.—C. S. Kittredge, M. D., of Oakland, Cal. (*The Western Lancet*), late Assistant Physician at the Nursery and Child's Hospital, Randall's Island, N. Y., publishes 19 fatal cases of this formidable affection—which is a malignant form of ulcerative stomatitis—induced in children under five years of age, predisposed to tuberculosis. The duration of this disease is from six to sixteen days, running a most rapid course when commencing in the cheek or throat, but somewhat slower in its progress when commencing in the gums. The disease demands prompt and energetic attention. He commences with the chlorate of potassa, in from five to ten grain doses every four to six hours, and continues it during the whole progress of the disease. The mouth must be frequently washed with a weak solution of liquor sodæ chlorinatæ, ℥ i. to water ℥ xii., and after mortification has commenced, a pledget of soft linen, wet in this solution, should be constantly kept between the sore and the adjacent tissue. Tonics and stimulants should be freely given on account of the great prostration, and iron with bark or quinine in as heavy doses as the child will bear, and strong beef tea in place of solid food. Out of 67 cases of stomatitis treated during the year, there were 19 deaths, or 28 per cent. Of the fatal cases, 12 were males and 7 females. The average age was 2 years and 11 months. In 16 cases, the primary disease was rubeola. Fourteen autopsies were made; and in every case, tubercles were found in great abundance. In 13 cases the tubercles were in the lungs, and in the other, the mesentary was filled with miliary tubercles. He remarks in conclusion that cancrum oris has a close connection with tuberculosis; and believes that it can exist only in children of a tuberculous diathesis.—*The Medical Record.*

Veratria in Heart Disease.—Dr. Bitot, of Paris, lately read a paper on the employment of veratrine in cardio-vascular affections not yet arrived at the period of cachexia. In consequence of experiments in the laboratory, and clinical observations, M. Bitot has been able to determine what is the mode of action of veratrine; and comparing these results with similar ones obtained with digitalis, he has deduced the following conclusions:

Veratrine is a precious agent for vascular troubles, more particularly for those troubles which accompany functional hypertrophy of the heart. Contrary to digitalis, in physiological doses it is, with regard to the heart, atonic and hyposthenic. In physiological doses, it is not spoliative as digitalis. Continuing its use has not, then, the same dangers. Its use seems to be as an indirect compensator. In superexciting the sensibility and contractibility of animal life, it causes morbid activity of the nervous system and the contractile fibres of vegetable life. Its action is very distinct from that of digitalis. When the latter is ineffectual, the former may be appealed to as well as digitalis. Veratrine is contraindicated in the last period of cardio-vascular affections and in asystole. Finally, there is ground for trying it in all the diseases which affect the nervous system and vegetable life.—*Med. & Surg. Reporter.*

ON THE TREATMENT OF CHRONIC ULCERS.—Dr. Henry Wagner, of Sidney, Ohio, says:

Not a little annoyance is often experienced by physicians in treating chronic ulcers. Almost all plans of treatment have proven beneficial in some cases, but with more or less disappointment to the physician. The treatment I have found most beneficial in all kinds of chronic ulcers, is the local application of iodine, applied to the entire surface of the ulcer, with tonic and alterative treatment. I have been more than pleased with the result of fifteen cases, that have recovered under this treatment within the past two years. Three of these cases were patients over fifty years of age, and had suffered from the ulcers more than twenty years; but I am happy to say they yielded at once on applying the iodine, with very slight pain to the patient; and by the use of ferri muriatis tinctura gtts. xx. three times a day they were speedily cured. The other cases were persons of broken down constitution and enfeebled health generally.

One, a lad fifteen years old, had an ulcer on his shin of right leg. The ulcer covered the entire front and extended down the leg about five inches; it presented a very unhealthy appearance, with ragged edges, and was quite offensive. He was unable to walk, and experienced great pain, but on applying the iodine the pain subsided. In three weeks' time he was able to walk, his ulcer healed in five weeks, and is now perfectly well.

The iodine sets up a healthy action in the morbid tissues of the ulcer, a free and more perfect flow of blood circulates through the morbid tissues, the ulcer takes on healthy granulation, and a new skin is formed. The scab formed by the iodine on the ulcer should never be removed until it comes off itself. The ferrum and potassium change the blood and build up the broken down constitution, and a happy cure is the result.

Hoping these few cases will be sufficient to show the efficacy of the iodine applied locally to chronic ulcers, I will not mention in detail other cases of equal interest and success. I only bring these few cases forward in order that those who have more opportunities of treating chronic ulcers, may be tempted to try the iodine, and I hope the results may be as satisfactory in their practice as they have been in mine. I feel very sanguine concerning its efficacy. I only ask those who make use of it, to be kind enough to publish their cases, whether attended by success or otherwise.

TO KILL LICE.—The Edin. Med. Journal for Nov., 1872, says that all kinds of lice and their nits may be got rid of by washing with a simple decoction of stavesacre, or with a lotion made with the bruised seeds in vinegar, or with the tincture, or by rubbing in a salve made with the seeds and four times their weight of lard very carefully beat together.

Over 80,000 persons are said to have died of cholera in Russia during the past year.

Uses of Chloride of Ammonia.—Dr. John Dewar, in the *British Medical Journal,* has the following remarks on this drug: "I consider it a very useful remedy; but, because of its very disagreeable taste, as well, perhaps, as on account of its being an old (and therefore apt to be forgotten) drug, it has not been used internally so much as its usefulness would seem to indicate. Its diaphoretic action is equal to its diuretic. It has been for a long time used more or less, especially on the Continent, in ascites. But it appears to have a special action on serous membranes generally; and I have found it very valuable in effusion into the pleura, especially when its cause is of a subacute or chronic character. Some time ago, I recorded a case of chronic hydrothorax where the effusion was very abundant, and occurring in a delicate woman, who ultimately died of phthisis. Under the use of the drug, rapid absorption took place. Since then, I have tried it in one or two other cases of similar nature—the effusion resulting from subacute pleurisy. On of the cases occurred in a scrofulous boy, the whole of whose left chest was filled with fluid, which rapidly disappeared after taking the chloride in fifteen-grain doses every four hours. It is necessary to give to adults from twenty to thirty grains every three or four hours, in order to get its full benefit. Its precise *modus operandi* has not been clearly ascertained. Although primarily not a stimulant, it may act on serous membranes by stimulating their power of absorption. Its diaphoretic action may account for its efficacy in muscular rheumatism, in which affection I have used it very extensively, and have found it to give more relief than any other remedy I have tried. I have had a personal experience of its good in muscular rheumatism coming on after scarlet fever. In these cases, also, it requires to be given in half-drachm doses. I have not found it of much use in lumbago or articular rheumatism. Some bitter infusion is the best thing to disguise its taste. I tried it mixed with sugar; but that, though it disguises its salt taste, makes it rather nauseating. With Dr. Thompson, I think this drug is deserving of further trial in other diseases, as well as in those above mentioned."

Treatment of Varicose Ulcers.—A. J. Steele, M. D., of St. Louis, Mo., (Med. Archives, October, 1872), speaks with confidence in the treatment of these ulcers, of that method first proposed by Mr. John Scott, formerly surgeon to St. George's Hospital, after an uniform success in twenty-five cases. The plan, though modified in detail, still retains the author's central idea, which was—equable support to the vessels of the entire foot and leg, and especially of all that part below the ulcer. Nothing accomplishes this end so efficiently as the properly applied adhesive plaster. Bandages alone have not the firmness; they loosen and become relaxed. It is essential that the plaster be evenly applied—no folds or creases, no uncovered angles or spaces—otherwise there will result irritation, pain, excoriation. After the strapping he applies a flannel bandage from five to seven yards in length, and about two inches in breadth. The entire foot and leg to the knee should be included.—*N. Y. Med. Rec.*

Local Uses of Tannin.—Dr. G. P. Hachenburg (N. Y. *Med. Record,* Aug. 15, 1872) reports several cases of the use of this remedy in prolapsus uteri, where other means had failed to afford relief. His method is as follows: A glass speculum is introduced into the vagina so as to push the uterus into its place. Through the speculum a metallic tube or syringe, with the end containing about thirty grains of tannin, is passed. With a piston, the tannin is now pushed against the uterus, the syringe withdrawn, and the packing neatly and effectually completed, with a dry probang, around the mouth and neck of the womb. After the packing is completed, the probang is placed against the tannin, in order to hold it, and the speculum is partially withdrawn. The packing is now fully secured and the instrument removed.

The application of tannin holds the uterus firmly and securely in place, not by dilation of the walls of the vagina, but by corrugating and contracting its parts. At first, the applications may be made weekly, finally but once or twice a month. It not only overcomes the hypertrophy and elongation of the cervix, but even, the writer thinks, induces a slight atrophy of the parts.

As a remedy for leucorrhœa, where the seat of the inflammation is at the mouth of the womb, or within the vagina, it actually gives speedy relief.

Dr. H. also reports a case of chronic ulceration of the rectum, which was cured after a few weekly packings of tannin.

He has found, moreover, that in affections of the throat, direct applications of tannin to the diseased parts give satisfactory results. In a case of extraordinary hypertrophy of the tonsils, preparatory to the operation of extirpation, tannin mixed with tincture of iodine to the consistency of syrup, was applied with the effect of so diminishing the hypertrophy that a surgical operation will in all probability not be necessary.

No remedy has given such satisfactory results in certain forms of chronic ophthalmia and opacity of the cornea as tannin. Once a week, place under the eyelids pure, well-triturated tannin. The application is not very painful, and the tears soon dissolve the tannin. An aged lady, who had chronic ophthalmia, was relieved by one application; another, who was blind from opacity of the cornea and chronic ophthalmia, recovered her sight mainly from the local use of powdered tannin.—G.—*Boston Med. and Surg. Journal.*

Spasmodic Asthma.—Nitrite of Amyl is considered very good, but the prescription ordinarily used is a drachm of cp. spts. of ether with ten minims of tr. of belladonna three times a day. This mixture may be continued for weeks, and many patients improve markedly under its influence.

Nevada Soda.—A small lake in Churchill county, Nevada, covering an area of seven acres, is a perfect well of carbonate of soda in its almost pure state. Twenty thousand tons a year can be obtained from it. It is pronounced by chemists equal to the European soda.

Treatment of Phthisis.—The general therapeutics in these cases are such as the profession are already familiar with. The only new remedy which has been employed for the relief of the night-sweats is the tr. of belladonna, given in ordinary doses, once or twice a day. This remedy has given very satisfactory results in all cases in which it has been used.

For the purpose of subduing the fever, which in some cases becomes the most prominent symptom for treatment, Heim's pill has been used with excellent results:

℞—Pulv. herb. digitalis.......................℈ ss.
Pulv. rad. ipecac..........................
Pulv. opii. puri.........................aa gr. v.
Fxtract helenii.......................... q. suf.
Pill No. xx. Consp. pulv. rad. ind. flor.
S. a pill three times a day.

To control the diarrhœa which sometimes appears, salicine has been used with better success than any other remedy which has been employed. It is given in ten-grain doses every four hours, either in the form of a pill or powder, as most agreeable to the patient. What is equally satisfactory is, they have remained cured. Salicine has therefore come to be one of the methods of treatment for chronic diarrhœa, whatever cause it may be dependent upon, or with whatever disease it may be associated. There are occasional cases which do not respond to this remedy; but in general, the results have been far more satisfactory than with the use of any remedy.

Physical Exercise in the Treatment of Invalid Women.—Dr. Frank A. Ramsey, in a communication to the Gynæcological Society of Boston, says: "In very many hundred females that have passed under my professional observation during the past thirty years, I have had no difficulty in destroying all the inconveniences of ordinary displacements, by the development of the muscles of the abdominal walls, and all the muscles that could be, by voluntary use, that have any connection with the sexual organs."

Dr. Storer remarked, that this question was one of much practical importance. He did not believe that active or passive exercise of any muscles, or system of muscles, could of itself cure displacements or other forms of uterine disease, as had been claimed by some; though indirectly, by improving the general bodily condition, it might effect a great deal of good. He had tried the health lift himself, and had recommended it to others. He believed that it not only was provocative of better rest at night, but it seemed often to prevent nervous headaches, where that tendency existed, and it was claimed that when such a headache was present, it might be cut short by heavier lifting than usual. The theory of the lift was that under its influence the circulation was equalized. Congestion, external and internal, were, to a certain extent counteracted and the activity of the capillaries increased.—*Detroit Review of Medicine.*

CHOLERA INFANTUM.—Dr. Lueck states that in the treatment of this disease he tried almost everything recommended by good authorities, sometimes to his utmost disappointment. Subnitr. of bismuth and calomel have never shown any good effect in cholera infantum under his employment, hence he has now discarded these remedies altogether in the management of these cases.

From the different opinions expressed to-day, the Doctor thinks we may safely conclude that the true pathological nature of this disease has not yet been demonstrated. However, it is generally concluded that cholera infantum is only a *functional derangement of the digestive system.* In fact he looks upon it as a *catarrh* of the *stomach* and *alimentary canal.* Fermentation, and not chymification, is going on the stomach and duodenum, and the vomiting and purging is only the result of this lactic fermentation. Hence, to prevent fermentation and promote normal digestion, would be the indications for treatment. These indications can generally be accomplished in a recent case by a few doses of the spiced syrup of rhubarb. If, however, the disease has lasted for some time, this simple remedy has no effect, and then we have found the following combination of great value:

| | | |
|---|---|---|
| ℞. | Boudault's pepsine, | gr. ij. |
| | Creasote, | gtt. 1-8 |
| | Opium pulv., | gr. 1-10 |
| | Acid tanic, | gr. 1-6 |
| | Ol. cinnamoni, | gtt. 1-8 |

For a dose to a child one year old, and repeated every three to four hours.

Here we have the creasote and pepsine to check fermentation; opium and aromatics to allay irritation, and again pepsine with tannin to give tone to the relaxed digestive system.

These few remedies, combined in various proportions to suit the circumstances of the case, we have used almost exclusively for the last three years, with a very satisfactory result.

However, in chronic cases, and in those children who are brought up by the hand, fermentation seems to go on faster than these remedies are able to check it; hence we must discontinue all milk-diet and give antiseptic nourishment. This we have prepared in the following manner: The yolk of one egg is beaten up well with one teaspoonful of loaf sugar; afterwards adding gradually 8 oz. of water. This quantity is given at short intervals during the twenty-four hours. Along with this food thin oatmeal gruel is allowed as a drink in place of water. As soon as the more active symptoms have past, gruel, or arrowroot is allowed more freely, boiled thoroughly to the consistency of syrup. Thus prepared, it has been an excellent article of diet for this class of patients, notwithstanding authority teaches us that farinaceous substances cannot be digested by children. With this treatment counter-irritation is combined in the form of hot baths and sinapisms.

There is a form of cholera infantum characterized by heat of the head, flushed face, and frequent purging, with little or no vomiting, which seems to depend on a catarrh of the small intestines without implicating the stomach. In this affection opium causes congestion of the brain, and cannot, therefore, be used; pepsine generally augments the intestinal irritation, and must, therefore, be left out of the treatment. Alkalies with small doses of ipecac first, and afterwards mild astringents, have done excellent service in these cases.

Acetate of Potash and Iron.—In the treatment of the diseases arising from defective nutrition in children this preparation is worthy of more consideration than it at present receives. Strumous diseases in this class of patients are always troublesome to cure. The iodide of potash has been the main dependence with many practitioners. The acetate will be found a good substitute for it.

Strumous opthalmia not unfrequently yields to this treatment, and uniting the acetate of iron with it, we obtain as good a formula as can be desired. Especially is this the case when the urine is of high specific gravity, loaded with lithates, the belly large and tumid, the limbs emaciated, the child fretful and peevish, appetite variable, bowels alternately costive and loose, the body anæmic, the sleep disturbed by dreams, and the breath offensive.

Combining the acetates of potash, ammonia and iron, we can accomplish great results at times after we have been perplexed, baffled and discomfited in our efforts with other means. When the lymphatics are large and knotty, this mode of treatment accomplishes a great deal of good.

At times the acetates have to be carried up to full doses before any sensible good is derived from them. It is of great utility, during the while, to give the child some preparation of the cinchona bark—say the fluid extract—or compound tincture, made of four times the strength of the officinal, so as to avoid so much of the alcohol being taken. This combination of the tonic properties of the cinchona, along with the acetates, will be found very excellent in all those refractory cases alluded to above. A good formula for making the acetate of iron is: Dissolve the recently precipitated carbonate of iron in strong acetic acid up to saturation. I believe that the acetate of iron is the most desirable ferruginous preparation in many forms of infantile affections; at least, in my hands it has given the best results. We will often be gratified by the steady mending that is established by this simple treatment in a very resistant class of disorders.—*Medical Archives.*

Pulse of Various Animals.—Vatel, in his "Veterinary Pathology," gives for our domestic animals the following pulse: Horse, from 32 to 38 pulsations per minute; ox or cow, 25 to 42; ass, 48 to 54; sheep, 70 to 79; dog, 90 to 100; cat, 110 to 120; rabbit, 120; guinea-pig, 140; duck, 135; hen, 140.

The number of physicians residing in and about Boston, is 339.

Household Remedies.—Dr. J. Henry Carstens, (Detroit Review of Medicine,) writes: How we often smile when red flannel is mentioned. Why not use white? The annodyne properties of the cochineal with which the flannel is colored, will account for this. At present, probably no cochineal is used in the dying process. Ink is no elegant preparation to apply to the skin in ring-worm, still the astringents, iron or tannin, in the writing fluid are most efficacious in curing herpes. Charcoal is very useful in the diarrhœa, of typhoid fever. For night sweats, as well as for other excessive perspirations, sage (salvia off.), is very serviceable. Horehound is an expectorant. Asthma is often greatly relieved by the application to the chest of a decoction of tobacco. Country people successfully use pumpkin seeds (cucurbita pepo.) in painful micturition, and to destroy tapeworms. An only too well known emenagogue is the common yarrow (achillea millifolium). Decoction of oak bark is a very good injection for the symptom leucorrhœa. Even the use of burnt sponge for scrofula has some good foundation, as sponges contain iodine. Sassafras thins the blood, is therefore good for plethora. Medical men in different parts of the country have again called our attention to the efficacy of wild indigo (baptisia tinctoria) in tympanitis, from whatever cause. The intense occipital headache of albuminuria can be mitigated by the Indian hemp (apocynum cannabinum), a plant growing in almost every field. Larkspur seeds (delphinum staphisagria) will destroy lice and other vermin that prey upon the human body. The common elder flowers (sambucus can.) act signally in deficient perspiration, very useful in gastric fevers; this remedy is also highly recommended in Bright's disease. How soothing is a dry hot poultice made of hops in facial neuralgia. An infusion of chamomile is an appetizer, and a decoction will often cure the simple fevers of children. The mothers relieve their children's colic with catnep. Cloves stimulate the torpid bowels. Fennel or Anise seed are good for flatulency. Alum prevents excessive granulation, and also cures chilblains. We have no better remedy than borax for hoarseness. Rose leaves (contain tannin) are made use of in aphthous sore mouth. In chronic malarial toxæmia few remedies can compete with black ash bark (fraxinous sambucifol.) Lobelia (lobelia inflata) relieves spasms of the glottis, and also as frequently tetanus as any other remedy we have at present at our command. Old people relieve their painful ulcers with stramomium leaves. Chronic constipation can be more effectually treated by those pleasant and palatable desserts, tomatoes and Amercan rhubarb, or pieplant, than by other remedies; while the costiveness of newborn infants can be overcome by a teaspoonful of molasses (not syrup), a remedy the children never object to taking. My friend Prof. Geo. P. Andrews informed me that he had cured amenorrhœa (due to colds) with a decoction of pennyroyal (hedeoma puleg.) after other remedies had failed. So the pride-weed (erigeron canadense) growing on the roadside can cure gonorrhœa. This list might be extended, but this is sufficient to show that a little knowledge of botany and of these household remedies are a great aid to a physician, especially when practicing in the country. These remedial agents are especially useful in the diseases of children. Instead of ignoring them, it is better to inquire into their value; for our object must ever be to do all in our power to relieve the suffering and heal disease.

Rheumatism of the Urinary Bladder.—This man, 39 years of age, complains of pain in the groins and frequent micturition, which attacked him two weeks ago and is increasing. He passes his water, on an average, about twenty times in the twenty-four hours, and is compelled to get up at night sometimes twelve or fifteen times, for that purpose. The urine is never bloody, and he voids it in a very small stream with considerable straining, but without any particular pain at the time. He is troubled with some flatulence, constipation and hemorrhoids, but his general health is not much affected, and he says he would sleep well enough at night if it were not for his bladder. He informs us that he has had rheumatism, but not since this trouble commenced.

On passing the sound, we detect no stricture or calculus in the bladder to account for the symptoms; which in all probability are produced by rheumatism or neuralgia, as the bladder is subject to these affections just as any other of the body is. We will, therefore, give him ten grains of quinine morning and evening for five days, and: ℞. Morphiæ sulph., gr. $\frac{1}{8}$; vini colchici, gtt. xlv. M.; at bedtime, each night.

The morphia will relieve the irritability of the bladder, and the quinia and colchicum will act upon the rheumatism. If this is insufficient to procure for him good rest at night, without which his health cannot be restored or maintained, we might give opium or morphia suppositories at bedtime, or inject half a drachm of laudanum, or give morphia hypodermically, in addition. He shall be kept warm, in the house, and may eat light food; but coffee, pastry, and the red meats are interdicted.

One week later.—The patient reports himself as being much improved. He is now compelled to empty his bladder only seven or eight times in the twenty-four hours, and not oftener than four times in the night, instead of every half hour, as before. He has no pain in his side, and no scalding or tenesmus.

There is marked improvement here, and in a short time he will have fully recovered. A full dose of morphia or quinia will frequently accomplish more than a dozen smaller ones, on the principle that when a man is hungry he wants a full meal. We must give medicine to accomplish a certain object, and should always give enough to obtain its full therapeutic effect. We will now give him: ℞. quiniæ sulph. gr. x, morphiæ sulph., gr. $\frac{1}{8}$. M.; which he shall take at night as before; and to modify the urinary secretion, he may take: ℞. Infus. uvæ ursi, f ℥ ij; sodæ bicarb., gr. x. M. S. t. d.

He returned three days later, and reported himself comfortable, and almost entirely cured.—*Clinic—Prof. Gross—Phil. Med. Times.*

Cerebro-Spinal Meningitis.—Doctor G. S. Mitchell, (Medical Examiner, May, 15th, 1873,) writes: "In all cases where there are reasons to believe that the stomach and bowels are loaded with food, or an excess of bilious matter, I give a mild emeto-cathartic of calomel and ipecac, aa. ten to fifteen grains, and repeat in twenty or thirty minutes if emesis is not produced. If this does not produce purgation in a short time, give an injection of soap suds. Whilst this is being given apply extensively and repeatedly mustard to the whole length of the back, to the extremities, chest and abdomen.—These should be continued till the muscular contraction and delirium are relieved. Blisters act too slowly, and appear to increase the irritability. They may be used to better purpose after all the active symptoms pass off.

Immediately after the action of the emetic, I give, to a patient 12 years old, the following:

℞.—Sulph. quinia, grs. iii. to iv.;
Sulph. Doveri, grs. vi. to viii.;
Sulph. morphia, gr. $\frac{1}{8}$;
Calomel, grs. iii.; every two, three or four hours, according to effect upon the pulse, and restless condition of the patient. If there are no muscular contractions, and a tendency to coma or stupor, I omit the morphia.—Between each dose I give the following;

℞.—Elixir valerian ammonia, ʒ i.
Bromide potassii, grs. iii. to iv.
Tinct. gelsemium, gtt. xx. to xxx.
Carbolic acid, gtt. iii.

The doses of these remedies must be regulated according to their effects upon the patient. The pulse must be brought up full and strong, the muscular contraction and excessive irritability must be controlled. This can only be done by the combined effects of tonics, opiates and sedatives. Quinia alone will not answer; neither will sedatives and opiates. I give carbolic acid for its antiseptic effects. To withhold quinia and stimulants because the pulse may be from 80 to 110, and to withhold nervous and arterial sedatives because the pulse is below that point, is dangerous. Whiskey may be used in the early stage, as a nerve excitant, to aid in effecting reaction; further than this its use is dangerous, because it increases the irritability and delirium. It may possibly act as an antiseptic in the course of the disease. After producing one evacuation of the bowels, keep them controlled for 48 hours or longer—(an excessive run upon the bowels the first two days will tend to fatal results)—continuing the calomel in small doses, with a view to its constitutional effects. This will be a safeguard to effusion upon the membranes of the brain, if there should be such a tendency. The application of boilod ears of corn to the patient's body, if not used too extensively, will assist in relieving muscular contraction, and supplying the body with heat. It is too much of a depletory remedy to be used extensively. The application of dry heat is much better. After reaction has fully taken place, and the violent cerebro spinal symptoms are relieved, I give the following :

℞.—Tinct. colchicum, } aa. ss.,

Fl. ext. ergot, }

Iodide potassi, grs. iv., as a remedy for the rheumatic complication, still continuing the other remedies as long as there are any cerebro-spinal symptoms.

After the violent symptoms have passed off, the patients are left in a prostrate and debilitated condition, for which I have used with decided benefit elixir calisaya bark, with strychnia, hypophosphite, iron, lime, soda, and potassa, aa. one drachm, three or four times a day. They produce a decided vitalizing effect."

Treatment of Tremor by the Hypodermic Injection of Arsenic.—As tremor has frequent origin in affections of the spinal cord, and as arsenic has an elective action upon that part of the cord which is concerned in sensitive impressions, M. Eulenburg has resorted to the medication of tremor by arsenic in hypodermic injections.

The treatment is based upon the employment of the arseniate of potash or Fowler's solution by subcutaneous injection. In this way the stomach escapes the irritant action of the drug. Neither does gastralgia manifest nor anorexia.

M. Eulenburg employs for his injections a solution of one part of liquid arseniate of potash to two parts of distilled water. He injects a half a syringe full at a time, that is, about 20-24 ctg. of the liquid arseniate at a dose. This is a considerable quantity when we consider the activity of absorption after hypodermics with that of the stomach. The injections were made over the nucha or spinal column, and were never followed by abscess. The pain produced is very light, but greater than that after the hypodermic use of morphia. The author has treated seven cases, now, in his way. In one of them he made 28 injections in 17 days. At first the tremor was slightly increased, but the final result was satisfactory. In a second case tremor ceased after five injections. In one of two other cases, presenting great analogy to paralysis agitans, fifteen injections were required to check the tremor ; in the other case, a remarkable amelioration was manifest after four.

The author then cites other affections in which this treatment has been employed, but results have not been definite as yet. This medication is to be considered hence as still in the period of trial, but all observations go to prove the perfect innocuousness of arsenical injections.

Hypodermic injections of the arseniate of soda have been employed by M. Lehmann at Copenhagen in the treatment of pernicious puerperal fever, without any success, however. Later V. Graefe used them in the algide stages of cholera, and Lewin has employed them successfully in the treatment of psoriasis. Finally, Levin Smith has recently recorded good results from their use in chorea. — *Gaz. Hebdom—Lyon Med.—Clinic.*

Scarlet Fever—Treatment.—Dr. T. W. Egbert, of Oil City, (*Trans. Pa. State Med. Soc.*,) reports that in an epidemic in that place he treated as follows: "I discard the varieties as designated by our authorities, believing scarlet fever to be one and the same disease, in all places and under all circumstances, modified, of course, by atmospheric, hygienic, and other known and unknown influences. My treatment, from the beginning to the end of the epidemic, has been uniform, simple, and, no doubt, novel to many practitioners; but successful results will always speak for themselves. From the incipiency of the epidemic until this time, I have treated, as near as I can glean from my record, two hundred and seventy cases, with but a *single* death; and in that case, as Dr. Davis can testify, my directions were reversed by the nurse, who applied hot, instead of cold, applications to the throat. From the incipiency of the disease until the desquamation is perfect, I prescribe for the patient the following mixture:—

"℞.—Acid. muriatic., f ʒ j.

Syr. simplicis, f ℥ ij.

Potass. chloratis, ʒ iij.

Aquæ rosæ, f ℥ iv. Mix.

"Sig. Half tablespoonful every two hours.

"The dose designated in the above prescription would be for a child, six years of age, double the amount necessary for an adult, and smaller quantities for a younger child. Where there is much restlessness and nervous irritability, I administer paregoric in sufficient quantities to soothe the patient and allay those symptoms. I have not found it necessary, in a single instance to use gargles, probings, or the pencil to the *fauces* or throat. In one case, that of a male adult, æt. twenty-four years, married; confined to his bed, with the characteristic scarlet blush making its appearance on the face and neck; general symptoms all present in an aggravated form. Precribed as follows:—

"℞.—Acid. muriatic., f ʒ ij.

Syr. simplicis, f ℥ iij.

Potas. chloratis, ʒ iv.

Tr. opii camph. f ℥ j.

Aquæ rosæ, f ℥ iv. Mix.

"Sig. Tablespoonful every two, three, or four hours.

"This was the principal treatment until the twelfth day, when the febrile symptoms had all subsided and desquamation well advanced; with the exception of simple tonics, continued for ten days or two weeks longer, this was the entire treatment of this case, and in sixteen days from the first appearance of the blush, he was at his office, attending to his ordinary business, being an oil broker. The reader can judge of the severity of this case and the efficacy of the treatment, when I state that there were no bad *sequelæ*, except perfect *onychoptosis* of both hands and feet. In a few cases where there was much congestion about the *fauces* and throat, ulceration of *uvula* and *fauces*, and enlargement and induration of the parotid and submaxillary glands, I found it necessary to use the ice-bag, applied snugly to the throat and neck until relief was obtained, which was generally from six to twenty-four hours, being careful not to freeze parts by continuous application too long at a time."

On a New Mode of Treament of Functional Dyspepsia, Anæmia, and Chlorosis (*The Practitioner*, March, 1873).—M. Brown-Sequard, in his "Archives of Scientific and Practical Medicine," in a paper on this subject states that in 1851 he had to treat a bad case of dyspepsia, and succeeded in curing the patient by a plan of treatment which derserves attention, though it was long ago adopted by Dr. Watson in this country in cases of obstinate vomiting. M. Brown-Sequard has employed this plan with complete success in a number of cases of dyspepsia, of chlorosis, of anæmia, and also as a means of ameliorating or curing nervous affections caused by gastric disturbances or poverty of blood. In a number of instances where failure occurred it was found that the patients had not carefully followed the rules, and that the failure was, at least in a great measure, due to this lack of care. In two cases only some increase of flatulency and acid eructations took place during three or four days, when the plan was given up. In a case of dropsy, attended with anæmia, dyspeptic pains were increased for a week, when the plan was abandoned. The treatment consists in giving but very little of solid or fluid food, or any kind of drink at a time, and to give these things at regular intervals of from ten to twenty or thirty minutes. All sorts of food may be taken in that way; but during the short period when such a trial is made, it is obvious that the fancies of patients are to be laid aside, and that nourishing food, such as roasted or broiled meat, and especially beef and mutton, eggs, well-baked bread, milk, with butter and cheese, and a very moderate quantity of vegetables and fruit, ought to constitute the dietary of the patients under treatment. This plan should be pursued two or three, after which the patient should gradually return to the ordinary system of eating three times a day. M. Brown-Sequard's experience with the patients on whom he has tried the plan of feeding above mentioned, shows that the amount of solid food required by an adult is nearly always as follows: from twelve to eighteen ounces of cooked meat, and from eighteen to twenty-four ounces of bread. As regards the quantity of fluid he has always allowed, it has been notably less than the amount indicated by Dr. Dalton (three pints), and by Dr. E. Smith—four and one-half to five pints.

Curious Case of Criminal Abortion.—Dr. T. G. Thomas, in the American Journal of the Medical Sciences for April, 1873, records a case in which the wife of a physician used an umbrella wire 17½ inches long to produce abortion, wounding the right lung, and inducing death by pneumonia on the 16th day.

The ovary of a hen is said to contain about 600 embryo eggs.

ABSCESS OF MASTOID CELLS.—Dr. S. H. Dessau (N. Y. Med. Journal) reports the following case: Annie F., aged two years, was brought to my clinic at the New York Dispensary, August 2, 1871, to be treated for a puriform discharge from the left auditory canal, and from an inflamed opening at the upper portion of the temporal region, behind the auricle. The history that the mother gave was to the effect that an abscess had formed at the upper portion of the mastoid cells some weeks previous to her presentation at my clinic, accompanied with great pain and deafness, and had opened spontaneously at the point before mentioned, a week or so before I saw her. At the time, there was an offensive discharge of pus, indicative of bone-destruction. The child presented numerous features of the strumous diathesis.

A probe bent to a curve was passed into the opening over the mastoid cells, and made its exit at the meatus, bone being distinctly felt during its passage.

I placed the child upon the use of cod-liver oil, and injected Villate's lotion into the sinus, the injection escaping at the external auditory canal. This injection was repeated every other day, and in the mean time the mother was directed to use an injection of carbolic acid (20 drops to the ounce) twice daily.

I saw the child for several weeks, and then lost sight of it for many months, when I finally had an opportunity of securing the result of my treatment, by accidentally meeting the child, while inspecting tenement-houses for the Board of Health last summer. The sinus had healed completely, the discharge from the auditory canal had ceased, and the hearing on the affected side was almost perfect. This condition had existed for some time.

The following is the formula for Villate's lotion:

℞. Liq. plumbi subacet, ℥ j.
Cupri sulph.,
Zinci sulph., aa, ʒ iv.
Aceti, ℥ vij.

Dissolve the crystals in the vinegar, and add the lead slowly.

TREATMENT OF GONORRHEA BY TANNO-GLYCERINE PASTE.—Dr. Tomowitz, K. K. Regimentsarzt, Austrian army, reports the successful employment of Schuster's (Aix-la-Chappelle) tanno-glycerine paste in somewhat different form for syphilis and Gonorrhea. His formula is as follows: R. Tannini puri ℥ ss; Opii pulverisati, gr. iv; Glycerinæ p. sut ft. pasta.

Some 50-60 drops of glycerine are requisite to bring the paste to a proper consistency. A sound of elastic bougie is dipped into the paste, warmed over a stove or spirit lamp and thus smeared is introduced into the orificium penis to the fossa navicularis, where it is held for five minutes. This operation is repeated three times a day. In gleet, the catheter or bougie is carried back to the bladder and slowly withdrawn so as to bring the paste into contact with every surface of the urethra. Even in acute cases the pain is but very slight.—*Allg. Militrraztl. Zeit., August,* 11, 1872.—*The Clinic.*

Fuming Nitric Acid for Internal Piles.—Billroth records 26 cases of prolapsing piles treated by him in various ways. In four instances he applied the actual cautery, in ten the galvano-cautery, and in the remainder fuming nitric acid. The latter plan was pursued as recommended by Dr. Houston, of Dublin. The results proved eminently satisfactory. His mode of proceeding was as follows: A free evacuation of the bowels was obtained by means of castor-oil given the day previously. Before the operation the mass was brought down by an injection. The patient was then placed on the side with the knees flexed. The parts adjacent to the anus were first well protected by oil so that no injury should then be done them. A small piece of wood was then dipped in the acid and applied to the outside of the swollen mass, until it had become tolerably stiff and had assumed a yellowish-green color. It was then smeared with some simple form of ointment and returned within the sphincter. The operation was usually performed without an anæsthetic, and an opiate suppository was rarely given afterwards. It is proper to keep the patient in bed. Fever rarely follows, though retention of urine is not uncommon for the first few days. The eschar usually separates without loss of blood. It is proper to give castor-oil on the third or fourth day, provided no fæces have passed. Hemorrhage will be likely to occur if the fæces become hardened; such accidents, however, are readily controlled by ice. Of the patients treated in this way some were discharged on the 5th and 9th days, though severe cases were under treatment from six to eight weeks. Several of the patients were examined a year after the operation, and there was no stricture in any one of them.

Billroth believes that in very severe cases this treatment may fail, and then suggests the use of acid nitrate of mercury, as recommended by Curling.—*Wiener Med. Wochenscrift*, No. 35, 1871.

Nitrate of Amyl in Melancholia.—(Hostermann: *Wiener Med. Presse*, October 20, 1872).—In accordance with the suggestions from English sources and Prof. Meynert, of Vienna, Dr. Hostermann has made a series of experiments with this drug in cases of melancholy. Inhalations are preferable to subcutaneous injections or to the internal administration of the remedy.

In 10 to 15 seconds the pulse becomes more frequent; in 30 to 40 seconds the face becomes red, the conjunctiva injected, and the subjective warmth of the body increases. At the same the arteries pulsate more vigorously, the head feels constricted, and the patient experiences the feelings of mild intoxication after wine drinking. With these symptoms the patient is more lively, and shows it both in his features and his manner. The external signs of the drug disappear in three-quarters of a minute after the cessation of the inhalations; but there seems to be a somewhat more permanent change for the better in the psychical condition of the patient.

This treatment is not suited to those cases where the melancholia depends upon some cause of exciting anxiety; it is rather suited to those cases of melancholia due to sluggish circulation and impeded nutrition in the brain.

SULPHUROUS ACID.—Sulphurous Acid is a capital remedy. Given a moist, dirty tongue, neither pale nor red, in fever, and I prescribe Sulphurous Acid. The same condition in chronic diseases also indicates it. Add that peculiar condition of the papilæ of the tongue, that looks like bubbles from decomposing matter, and the indications are stronger. As a *gargle,* in sore throat, where the mucous membrane is tumid and relaxed, it has no superior. In such cases I prescribe it in the folowing proportion: ℞ Sulphurous Acid, ℥ ss. Water, ℥ ijss. For a gargle.

ANTISEPTICS.—Speaking of Sulphurous Acid brings up the class antiseptics, and it may be well to note the indications for their use —premising that they cannot be used indiscriminately. The more important of them are, Sulphite of Soda, Sulphurous Acid, Muriatic Acid, Chlorate of Potash and Baptisia. *Sulphite of Soda* is the remedy where the tongue is *pallid* and covered with a dirty coat.

*Sulphurous Acid* is the remedy where the color of the tongue is normal, but the coating is thick and dirty, and presenting the peculiar appearance of the papilla just named. A yellowish-red appearance of the fauces with fullness, is an excellent indication.

*Muriatic Acid* is the remedy where there is deep redness of the tongue, with dryness, the fur having a shade of brown.

*Chlorate of Potash* is the remedy where there is a bright redness or a shade of purple, but more especially where there is a cadaverous odor, or where we know the blood poisoning is from the absorption of animal matter. Thus it is *the* antiseptic in puerperal cases where a portion of the placenta, or the uterine excretions are undergoing decomposition.

*Baptisia* is the remedy where there is a dusky discoloration of mucous membranes with fullness.—*Cincinnati Med. Eclec. Journal.*

TWO NÆVI CURED BY MONSEL'S SOLUTION APPLIED EXTERNALLY.—Dr. Jacob Geiger, (American Practitioner),—writes:

A male child, aged nine months, had at birth a "mother's mark" on his perinæum and over the pit of his stomach. They were at first flat but slightly elevated spots, and quite small. When the patient was about six months old, however, the tumors took on a very rapid growth; that on the perinæum occupying not only the entire perinæum, but a portion of the scrotum also, while that on the abdomen was an inch in diameter. The perineal nævus was kept so constantly irritated by the child's diaper, his urine and his fæces, and having on more than one occasion bled considerably, I advised an operation for its cure. The mother positively refused her consent to any other procedure than one which consisted in some external application. I detemined therefore to try the methodical use of Monsel's solution to both the growths. Making a mixture of equal parts of the liq. ferri persulph. and glycerine, I painted not only the nævii themselves thoroughly with this, but I applied it also for some lines beyond, to the healthy skin, and directed it to be repeated twice daily. In a week both tumors had diminished appreciably in size; and in less than one month from the date of the first application of the iron they had disappeared altogether.

On Cholera Infantum.—Dr. J. H. Nowlin (Med. and Surg. Reporter,) writes; The fatality of this disease in city, town, and country imposes upon our profession the necessity and obligation of the most thorough investigation of its causes, the best mode of prevention, its pathology, and most successful plan of treatment. Omitting the causes, symptoms, and pathology of the disease, I will proceed to give the plan of treatment, which, after an experience of thirty years, has proven the most successful in my hands; premising, however, that I regard cholera infantum, when seen clearly, as one of the most readily curable of all infantile diseases.

1st. I enjoin strict rest in the horizontal position. 2d. The stomach having been evacuated by spontaneous emesis, as is almost always the case, I direct one to two grains of oxalate of cerium suspended in mucilage acaciæ after each effort to vomit. Should there be considerable prostration, with cold surface and extremities, I give brandy toddy in suitable doses, *pro re nata,* till the circulation and temperature become normal. Occasionally, as auxiliary, I direct the warm bath, given with the least possible handling. So soon as the stomach becomes quiet I direct the following: ℞. Bismuthi subnit. gr. xl. vel. xlviii. Ft. chart. No. xvi. vel. xx.

Sig. One powder in mucilage after each loose discharge (no matter how often) till the healthy interval is restored. No food or drink is allowed (mother's milk inhibited), excepting lime water and new sweet milk in equal proportions, given in ʒj to ʒiv every 15 to 30 minutes for the first 24 hours.

This course almost always secures the perfect quiet of the stomach, with rapid tendency to resume its normal functions, the bowels demeaning themselves in like manner. Nothing more is wanted as a general rule, but a cautious and gradual return to former prudent habits. I find no occasion for emetics, cathartics, or so-called, alteratives. In case of constitutionally feeble children, I generally direct a tonic to conclude the treatment.

Fluid Extract of Castanea Vesca in Pertussis.—Dr. Thomas S. Davis gives in the *Medical Times,* (Dec. 28, 1872), the results of the treatment of fifteen cases, with this remedy. The first eleven cases had the characteristic whoop, the remainder had well-marked paroxysms, but not the full spasm, and they recovered without having it. In each case the violence of the spasm was reduced even more markedly than the number of the paroxysms. The castanea was continued for a week, after which, in a few cases, a simple expectorant was given. The nurse in charge, who had witnessed many epidemics of the disease, declared she had never seen medicine act like it.

This medicine is made from the beans of the common chestnut tree, castanea vesca, natural order *Cupuliferæ.* The preparation used was the fluid extract made by Mr. John M. Maisch, of Philadelphia, (*see Amer. Journ. of Pharmacy,* Dec., 1871, p. 529). The dose is half a teaspoonful to a teaspoonful every three or four hours, for a child six years old.

THE WET SHEET IN THE ACUTE EXANTHEMATA.—The first effect of this proceeding—namely, of rolling the patient up in sheets wrung out of cold water, and surrounded by a woollen cover or dry sheet—is to powerfully excite the whole nervous system. Heat is withdrawn from the body in proportion as the temperature of the skin and that of the wet cloth approximate, and this again leads to a steady flow of the internal temperature toward the skin. If the body remain enveloped for a still longer period, so that the temperatures of the body and of the cloth have become equalised, a more or less abundant excretion of sweat occurs as a result of the cutaneous hyperæmia. On this increased excretion of sweat Steinbacher lays special stress, contending that by its means the special poison of the disease is eliminated from the body. In order to determine whether this supposition is correct, M. Hofman treated a child four years of age, suffering from a severe attack of the measles in the hydropathic fashion, but placed upon its chest a fine piece of linen; and after the child had lain for two hours in the wet cloth, and had perspired freely, the piece of linen was removed, and the sweat expressed from it received into small tubules. M. Hallier, at Jena, found the micrococcus abundant in the fluid, and at once instituted experiments, the results of which are not yet published, to determine whether this micrococcus will propagate the disease of measles. If this be found to be the fact, it will tend to show that the hydropathic plan of treatment is well adapted for the rapid removal from the body of the parasitic organisms. M. Hofman has adopted this method of treatment with good results in many severe cases of measles and scarlet fever, even when the patients were in the first instance comatose; and has observed not only that the febrile symptoms are rapidly subdued, but that convalescence of the patients is much quicker than under other plans of treatment.

MODIFIED OPERATION FOR PHIMOSIS.—Professor Sherman has practiced in a considerable number of cases, a modification of one of the old methods of treating phimosis, which differs sufficiently to be entitled to the name of a new operation. Instead of drawing forward the skin, with the intention of cutting off a circle of it at one sweep, as in the ordinary method, he seeks a greater precision by making a circular incision through the skin, near the base of the glands first, and then, by a second step, cuts off the mucus membrane close to its anterior edge. As a general rule, he does not slit up the membrane, but finds that it dilates with little force, and can be effected without the usual dorsal incision, so as to be stitched to the edge of the integument for union by first intention. The folded up edge of the mucus membrane thus becomes the new border of the prepuce, while the cicatrix is a delicate line further back, which occasions no inconvenience.—*Trans. Ill. State Med. Society.*

FOR CHAFING OF INFANTS.—Take of powdered starch two parts, white oxide of zinc one part. Make a fine, well-mixed powder. Dust the abraded places with the powder, after proper cleansing.

ON THE USE OF BATHS IN ACUTE RHEUMATISM, ATTENDED WITH HEAD-SYMPTOMS AND HIGH TEMPERATURE. — By Dr. Henry Thompson, of the Middlesex Hospital, (British Medical Journal, August 3, 1872.

A case has lately occurred in the Middlesex Hospital, illustrating the beneficial effects of the bath in acute rheumatism, attended with head-symptoms and high temperature. Dr. Thompson, in his remarks on the case, observes that it is only now and then, in a few rare and scattered instances, that acute rheumatism proves fatal by an unexpected outbreak of overpowering nerve-symptoms, and such a result would have ensued in the instance recorded, according to Dr. Thompson's opinion, if the bath had not been used. The fatal issue in similar cases was formerly referred to the occurrence of metastasis, meningitis, and the like, but now it is said to be due to hyperpyrexia. To this last term, if used in a practical sense, Dr. Thompson makes no objection, as the importance of very high temperature cannot be overrated as a symptom; but in a pathological point of view he thinks that its influence has been exaggerated, for the nerve-symptoms invariably precede the hyperpyrexia. Nevertheless a high body-heat, ranging from 108.6 degrees to 112, is incompatible with life, and it is necessary to lower it by such means as are available, and Dr. Thompson thinks that the use of the bath is the best therapeutical agent. The temperature of the bath is 90 to 95 degrees in the first instance, and is gradually reduced by the addition of cold water to 70 degrees.

The clinical thermometer must be practically the best guide for the employment of the bath, for at a lower body-temperature than 102.5 degrees it would not be desirable to use it. It is important to observe that, in the case recorded by Dr. Thompson, the most severe and extensive chest-complications, such as pneumonia, pleurisy, bronchitis, and pericarditis, underwent no perceptible change for the worse in consequence of the bath. The case itself is recorded at length, with the thermometrical observations taken at frequent intervals from day to day, and the effects produced by the baths, which were eight in number, are accurately noted. The case, however, although terminating in recovery, was a tedious one, and the convalesence was exceedingly slow.—*Med. Times.*

INFLUENCE OF BELLADONNA ON SWEATING.—In some interesting communications to *The Practitioner*, Dr. Sidney Ringer brings forward an abundance of evidence to prove that belladonna and its active principle are able to check and prevent sweating, whether the result of disease or induced by exposure to an elevated temperature. In the former case, his observations enabled him to conclude that one two-hundreth of a grain of atropia injected under the skin is generally sufficient to check sweating for one night. This dose produces a dryness of the fauces, but does not dilate the pupils. Stramonium, it was found, is able to exert the same influence.

The census of France for 1872 shows the population to be 36,102,921; a decrease of 366,935 since 1866.

