# Errata

**Published:** 1873-10

**Authors:** 


*Errata.*

In the article on "Tape Worm," page 610, in the prescription, oil of cinnamon should read: gtt. ii.

